# Maximum (prior) brain size, not atrophy, correlates with cognition in community-dwelling older people: a cross-sectional neuroimaging study

**DOI:** 10.1186/1471-2318-9-12

**Published:** 2009-04-02

**Authors:** Susan D Shenkin, Carly S Rivers, Ian J Deary, John M Starr, Joanna M Wardlaw

**Affiliations:** 1 Geriatric Medicine, Division of Clinical and Surgical Sciences, University of Edinburgh, 51 Little France Crescent, Edinburgh, EH16 4SB, UK; 2 Division of Clinical Neurosciences, University of Edinburgh, Western General Hospital, Crewe Road, South Edinburgh, Edinburgh, EH4 2XU, UK; 3 Department of Psychology, University of Edinburgh, 7 George Square, Edinburgh, EH8 9JZ, UK; 4 The University of Edinburgh Centre for Cognitive Ageing and Cognitive Epidemiology, Edinburgh, UK; 5 Clinical Trials Research Unit, University of Leeds, 17 Springfield Mount, Leeds, LS2 9NG, UK

## Abstract

**Background:**

Brain size is associated with cognitive ability in adulthood (correlation ~ .3), but few studies have investigated the relationship in normal ageing, particularly beyond age 75 years. With age both brain size and fluid-type intelligence decline, and regional atrophy is often suggested as causing decline in specific cognitive abilities. However, an association between brain size and intelligence may be due to the persistence of this relationship from earlier life.

**Methods:**

We recruited 107 community-dwelling volunteers (29% male) aged 75–81 years for cognitive testing and neuroimaging. We used principal components analysis to derived a 'general cognitive factor' (g) from tests of fluid-type ability. Using semi-automated analysis, we measured whole brain volume, intracranial area (ICA) (an estimate of maximal brain volume), and volume of frontal and temporal lobes, amygdalo-hippocampal complex, and ventricles. Brain atrophy was estimated by correcting WBV for ICA.

**Results:**

Whole brain volume (WBV) correlated with general cognitive ability (g) (r = .21, P < .05). Statistically significant associations between brain areas and specific cognitive abilities became non-significant when corrected for maximal brain volume (estimated using ICA), i.e. there were no statistically significant associations between atrophy and cognitive ability. The association between WBV and g was largely attenuated (from .21 to .03: i.e. attenuating the variance by 98%) by correcting for ICA. ICA accounted for 6.2% of the variance in g in old age, whereas atrophy accounted for < 1%.

**Conclusion:**

The association between brain regions and specific cognitive abilities in community dwelling people of older age is due to the life-long association between whole brain size and general cognitive ability, rather than atrophy of specific regions. Researchers and clinicians should therefore be cautious of interpreting global or regional brain atrophy on neuroimaging as contributing to cognitive status in older age without taking into account prior mental ability and brain size.

## Background

Ageing affects both brain volume and cognitive ability in non-demented older people. Brain volume declines with age: autopsy studies estimate around 2–3% per decade from around age 40 years [1]; [2], whereas neuroimaging studies estimate around 5% per decade [3], with changes being non-uniform across brain structures [4]. Changes in cognitive ability with age are generally described for two main components of intelligence: (a) crystallised abilities e.g. general knowledge, vocabulary, which are relatively well-preserved even into early dementia, and (b) fluid abilities, which tend to decline from early adulthood. Fluid-type abilities typically require abstract reasoning, particularly under time pressure, with new materials, where previous experience provides no advantage [5].

Over the last century several studies investigated the relationship between brain size and intelligence [2] in young adults. Before accurate measures using neuroimaging techniques were possible, head size was used as a proxy for brain size, and there was a small, but statistically significant, association between head size and intelligence: in adults a mean correlation of around 0.2 [6]. Head size is closely related to brain size, but these cannot be seen as equivalent. The size of the skull vault reflects the maximum size of the brain, and is attained by around age six years [7]. In older people, brain size (but not head size) decreases with ageing-related cerebral atrophy. Head size therefore reflects maximal, rather than current, brain size. In older people there is an association between head size and cognitive ability (r = .07 to .21) [8,9], but these studies did not account for prior cognitive ability. This is important because differences in cognitive ability in old age may be due to the stability of these differences from earlier in life, rather than a decline due to age. About 50% of the inter-individual variation in cognitive ability is stable from age 11 to almost 80 years [5].

Neuroimaging allows an in-vivo, non-invasive measure of actual whole brain volume that is a current and much more accurate measure of actual brain size than proxy measures such as head size. A meta-analysis of *in vivo *brain volume and intelligence reviewed 37 independent samples (n = 1,530), and found a correlation between brain volume and intelligence of 0.33 [10]. Twenty-four of the studies were in adults (r = .41 for females, r = .38 for males, .33 for sexes combined), but the mean age was not reported. Therefore, in neuroimaging studies, there is a consistently documented moderate correlation between brain size and cognitive ability in young adulthood but few studies have investigated whether this relationship persists into older age. Clinical studies in older people often comment on atrophy seen on neuroimaging, and it may be assumed that that 'bigger is better': that individuals with significant atrophy will perform less well [11]. However, the results of studies of brain size and cognition in older people do not confirm this. One review [12] suggests that "When structure-cognition associations are found, they are not easily replicated and appear sensitive to the sample composition and choice of cognitive measures." (p. 736)

One study that investigated the relationship between brain size and cognition in older adults measured intracranial area (ICA) – an estimate of maximal brain size [13] – and several regional brain volumes in 97 unmedicated healthy older men (mean age 67.8, SD 1.3 years). Regional brain volumes correlated with tests of premorbid and fluid intelligence and tests of visuospatial memory (r = .20 to .32) [14]. The relationships between specific cognitive tests and regional brain volumes were best summarized by a significant positive relationship between the latent traits of a general brain size factor and a general cognitive factor (*g*) (structural equation modelling, correlation = .42) and not by associations between individual tests and particular brain regions. Therefore, in healthy elderly men, the relationship between brain region volume and cognitive ability may be largely due to longstanding associations between general cognitive ability and overall brain size.

In a cross-sectional community survey in Australia [15], MRI scans were performed on 446 individuals (52.2% male, aged 60–64 years) who performed cognitive tests in addition to giving details of education, health and well-being. There was a correlation between cognitive ability (both crystallised and fluid) and both whole brain volume (WBV) and intracranial volume (ICV, a measure of maximal brain size) for men (r = .15 to .18) but not women (r = .02 to .06). There was no association between cognitive ability and a measure of atrophy – WBV corrected for ICV ((WBV - ICV)/ICV) – for men or women (r = 0 to -.1).

Only one study to date has published data on the relationship between brain size and cognitive ability in older people aged over 75 where cerebral atrophy is more likely to have an influence. In 92 individuals aged 79 (57.6% male) from Aberdeen, UK, there was no statistically significant association between total intracranial volume and cognitive ability (η^2 ^= .009) [16]. There is therefore a need for further studies, with information about prior and current cognitive ability and brain size, to investigate the relationship between cognitive ability and brain volume in both men and women over age 75.

In summary, ageing is associated with both generalised brain atrophy, and changes in cognitive ability – a relative preservation of crystallised-type and decline in fluid-type intelligence. Brain size is associated with cognitive ability in adulthood, and it has been suggested that the relationship between brain size and cognitive ability in old age is due to the persistence of this relationship from earlier life. Studies investigating the biological basis of ageing have often been cross-sectional, and have reported associations between regional brain volumes and specific cognitive domains in older people [12]. These are often interpreted as meaning that ageing-related atrophy in a particular brain region is associated with deterioration of a specific cognitive ability [17]. However, the association between brain structure and cognitive ability may originate earlier in life and remain stable over the lifespan [14]. Moreover, this association may be between overall brain volume and general cognitive ability, rather than between regional brain volumes and specific cognitive domains [14,18]. Studies of the structural basis of cognitive ageing therefore need to consider both maximal and current brain volume; both prior (typically using a valid estimate) and current cognitive abilities; and both specific and general cognitive abilities.

We therefore investigated the relationship between brain size and cognitive ability in community-dwelling older people (aged 75–81). We hypothesised that: (a) current brain volume would be associated with current cognitive ability, (b) this association would be attenuated when corrected for maximal (prior) brain volume and (c) the association would be due to the relationship between general cognitive ability and overall brain size, not specific cognitive domains and brain regions.

## Methods

### Subjects

We recruited 115 independently-living volunteers aged 75–81 years from the community. The majority responded to local advertising or media appeal, and some were invited by letter as part of the Lothian Birth Cohort 1921 study [19]. Volunteers with illness that would prevent participation, a diagnosis of dementia, or contraindications to MRI (identified by themselves or their general practitioner), were excluded [20]. All participants gave written informed consent. The study protocol was approved by Lothian Regional Ethics Committee.

### Cognitive testing

As described previously [20] we used the Mini-Mental State Examination (MMSE) [21] to screen for cognitive impairment: those scoring less than 24 were excluded. Subjects also performed the Controlled Word Association Test (Verbal Fluency) [22]: a test of executive-type function, Moray House Test No. 12 (MHT) [23], a test of verbal ability, Raven's Standard Progressive Matrices (RSPM) [24]: a non-verbal reasoning measure of fluid-type intelligence, and the Logical Memory (LM) subtest of the Wechsler Memory Scale [25]: a measure of immediate and delayed memory. We estimated prior cognitive ability using the National Adult Reading Test (NART) (Nelson and Willison, 1991): the ability to read irregularly pronounced words.

### Magnetic Resonance Imaging (MRI)

We used a General Electric Signa LX 1.5T scanner for standard structural imaging (axial T1-weighted spin-echo, T2-weighted and FLAIR fast spin-echo) and a coronal three-dimensional fast spoiled gradient echo T1-weighted whole brain volume sequence (TI 400 ms, flip angle 20°, slice thickness 1.7 mm, no interslice gap, FOV 24 cm, matrix 256 × 256) [20].

### Image analysis

**Figure 1:**
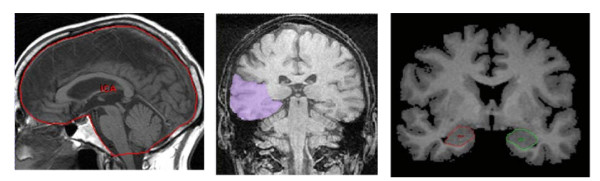
**Illustration of the definition of a) Intracranial area b) Temporal lobe volume c) Amygdalo-hippocampal complex volume**.

We used Analyze™ software (Mayo Clinic, Rochester, MN) on Sun workstations. We applied an intensity threshold separating the brain from the meninges for semi-automated analysis. *Intracranial area (ICA) *(a measure of maximal brain volume) was measured by manually tracing the inner table of the cranial vault on the midline slice of the sagittal localizer [13] (Figure 1a). *Whole brain volume (WBV) *was measured from the volume sequence coronal to the hippocampal long axis, including all brain tissue to a horizontal line across the inferior limit of the cerebellum [20]. *Frontal lobes *were measured separately, including the prefrontal lobe, from the slice in which the frontal pole could be distinguished from the meninges to the slice immediately preceding the genu of the corpus callosum. Measurements were made using automated methods with manual tracing to separate the lobes through the interhemispheric fissure; *temporal lobes *were measured separately including tissue from the anterior part of the temporal poles to the last slice in which the fibres of the crux of the fornix appears distinct from the hippocampus and the walls of the lateral ventricle (Figure 1b); *amygdalo-hippocampal complex volume *was measured bilaterally in coronal slices from the first slice in which the temporal stem was visible and the grey mass of the amygdala appeared enclosed by the medial temporal lobe white matter. In the initial slices, the amygdala was measured manually. As the slices progressed, the cortical amygdaloid nuclei were included as the medial boundary, and the lateral boundary was the temporal horn of the lateral ventricle. Superior and inferior boundaries were formed by the white matter of the temporal lobe, and were defined manually. Boundaries adjacent to CSF were defined by automatic edge detection using an autotrace seed. The hippocampus was defined as subiculum, hippocampus proper, and dentate gyrus with the alveus and fimbria. The last slice was that in which the full extent of the crus fornicis was visible following the recession of the pulvinar nucleus of the thalamus. (Figure 1d) [14]. *Corpus callosum area *was derived by manually tracing around the edges of the corpus callosum. *Ventricular volume *included the lateral, 3^rd ^and 4^th ^ventricles. The trained rater was blind to other data. To assess intra-rater reliability, volumetric analyses were repeated (n = 10) and showed errors of < 1%.

### Statistical analysis

**Figure 2:**
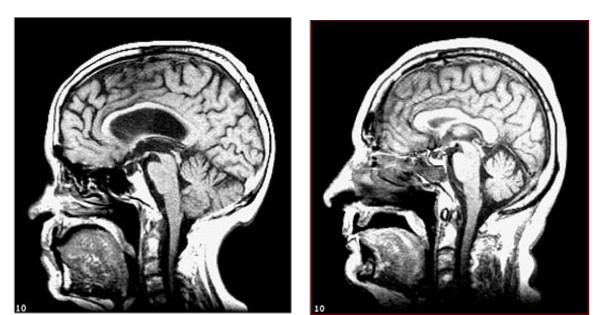
**Midline sagittal MRI scans to illustrate subjects with relatively more (a) and less (b) atrophy**. Atrophy is estimated by correcting whole brain volume (WBV) for intracranial area (ICA).

Sex differences between regional volumes were investigated using t-tests. Because there were sex differences in brain volumes, associations among cognitive tests and volumes were investigated using partial correlation (adjusting for sex). Years of education had a skewed distribution, therefore Spearman's rho was used to investigate the correlation between education and cognitive test score or brain volume. Partial correlation (adjusting for sex and education) was performed to assess potential confounding by education (to allow comparison with other studies; however, this also largely adjusts for prior cognitive ability). Cerebral atrophy was estimated by adjusting WBV for ICA, using standardised residuals from linear regression, and also by converting ICA to intra cranial volume (ICV) using the regression equation ICV = 5479.8 + (99.9 * ICA) [13] (Table 1), and estimating cerebral atrophy using the ratio (ICV-WBV)/ICV. Figure 2 shows examples of subjects with relatively high and low atrophy. Regional brain volumes were adjusted for ICA or WBV using standardised residuals from linear regression. Data reduction was performed using principal components analysis.

**Table 1:** Cognitive and neuroimaging results (volumes and area) for 107 subjects

**Variable**	**n**	**Mean**	**Min**	**Max**
**MMSE**	104	28.4	24	30
**NART (positive score)**	107	29.9	11	44
**RSPM**	104	30.5	12	51
**Moray House Test**	101	57.1	30	74
**Verbal fluency (total)**	107	37.1	15	78
**Logical Memory Total**	106	33.1	6	74
	**Mean**	**SD**	**Min**	**Max**
**Whole brain volume (cm^3^)**	1,135.5	98.4	947.4	1,405.2
**Intracranial area (cm^2^)**	148.6	10.3	129.3	173.7
**Estimated intracranial volume (cm^3^)**	1,489.8	103.0	1,297.1	1,740.5
**Corpus callosum area (mm^2^)**	540.0	83.5	392.0	775.2
**Ventricular volume (mm^3^)**	30,549.4	18,893.7	4,655.7	96,263.5
**Right frontal lobe vol (mm^3^)**	55,575.9	7,953.7	38,878.7	77,656.7
**Left frontal lobe vol (mm^3^)**	51,705.4	7,985.1	37,105.8	71,346.7
**Right temporal lobe vol (mm^3^)**	69,748.9	7,724.6	49,938.6	88,618.3
**Left temporal lobe vol (mm^3^)**	66,068.8	7,480.1	52,574.8	86,525.3
**Right AHC volume (mm^3^)**	5,056.2	706.0	3,609.9	7,122.6
**Left AHC volume (mm^3^)**	4,753.6	671.4	3,088.4	6,962.8

MMSE: Mini-Mental State Examination, NART: National Adult Reading Test, RSPM: Raven's Standard Progressive Matrices, AHC: amygdalo-hippocampal complex Cognitive tests n not all = 107 due to deafness, visual impairment, omission of part of test or timing errors.

## Results

### Descriptives

115 subjects were recruited, and 110 of these completed MRI. Of the completed MRI scans, 3 were excluded from analyses due to incidental findings (frontal meningioma, temporal cyst, pituitary adenoma). Therefore, analyses are presented for 107 subjects with valid scans: 31 (29.0%) male, mean age 78.4, SD 1.5 years. 49 (45.8%) subjects had a history of hypertension, 36 (33.6%) cardiovascular disease, 6 (5.6%) diabetes, showing that these subjects are comparable with other community-dwelling older people [4]. Subjects had a median of 9 years in full-time, formal education (range 7 to 22, interquartile range 9 to 11). Table 1 shows descriptive statistics for cognitive tests and brain volumes. There were no statistically significant differences between men and women on any cognitive test, or on years of formal education (P all > .22). Women had smaller volumes in all brain regions (t test P all < .001, except ventricular volume P = .04) except corpus callosum area P = .23. There was no evidence that the sex differences were attributable to differences in body morphology between men and women (correcting brain volumes for BMI, did not alter the sex differences [P all < .001 except ventricular volume P = .06]).

### Correlations

There were universally positive intercorrelations between the tests of current cognitive abilities (r .11 to .69). Using principal components analysis, the first unrotated principal component accounted for 51.9% of the total variance (factor loadings MHT .88, RSPM .83, VF .60, LM .50, cases excluded listwise, n = 99). Scores were retained for each subject on this 'general cognitive factor' (traditionally called g). There was no significant association between age and any cognitive test (r between -.14 and .11, P all > .26), or brain volume (r between .07 and .14, P all > .15).

The correlation between WBV and ICA was .79 (P < .01).

### WBV and cognition

There was a positive association between WBV and *g *(r = .21, P < .05) (Table 2). For specific cognitive tests there was a significant correlation between WBV and NART (r = .23, P < .05), RSPM (r = .27, P < .01), and MHT (r = .25, P < .05). The same pattern held for ICA and individual tests, and if correlations were corrected for sex.

**Table 2:** Correlations among cognitive tests and neuroimaging results (volumes and area)

	**WBV**	**ICA**	**CCA**	**VV**	**RFL**	**LFL**	**RTL**	**LTL**	**RAHC**	**LAHC**
**NART**	.23*	.30**	.13	.22*	.10	-.01	.15	.13	.19	.24*
**RSPM**	.27**	.25*	.23*	.04	.14	.01	.28**	.20	.10	.11
**MHT**	.25*	.28**	.21*	.08	.21*	.05	.29**	.16	.14	.15
**VF**	.15	.13	.15	-.01	.08	.03	.16	.15	.21*	.22*
**LM**	-.18	-.03	-.10	.07	-.02	-.10	-.18	-.21*	-.09	-.05
***g***	.21*	.25*	**.20**	.06	.16	.01	.23*	.14	.14	.16
***g *corr for NART**	.11	.11	.15	-.06	.13	.02	.18	.09	.05	.04

Partial correlation corrected for sex (df = 96). * P < .05 ** P < .01

n varies from 104 to 107 depending on cognitive test (Table 1) and n = 99 for *g*

WBV: whole brain volume, ICA: intracranial area, CCA: corpus callosum area, VV: ventricular volume, RFL: right frontal lobe volume, LFL: left frontal lobe volume, RTL: right temporal lobe volume, LTLV: left temporal lobe volume, RAHC: right amygdalo-hippocampal complex volume, LAHC: left amygdalo-hippocampal complex volume

NART: National Adult Reading Test, RSPM: Raven's Standard Progressive Matrices, MHT: Moray House Test, VF: Verbal Fluency, LM: Logical Memory

*g*: general cognitive factor (The first unrotated principal component of MHT, RSPM, VF and LM, accounting for 51.9% of the total variance: see text.)

*g *corr for NART: estimate of cognitive change (see text)

#### Education

Number of years of formal education correlated both with WBV (Spearman's rho = .26, P = .008) and ICA (rho = .35, P < .001), but with no specific brain region (rho >.14). Education correlated with performance on cognitive tests (NART rho = .41, P < .001; Ravens rho = .24, P - .015; MHT rho = .30, P = .002; VF rho = .10, P = .28; LM rho = .18, P = .06). Correcting for education attenuated the relationship between brain volumes and cognitive tests (WBV and *g *r = .18, P = .08; ICA and *g *r = .18, P = .08. Although the correlation between brain volume and cognitive tests no longer reaches conventional statistical significance, the correlation coefficients are not significantly different (for WBV and *g *falling from .21 to .18, and for ICA and *g *from .25 to .18).

### Correcting for maximal brain size (ICA)

Correcting for ICA attenuated the association between WBV and *g *from r = .21 to .03 (Table 3) attenuating the variance by 98%. There was no significant correlation between brain atrophy (WBV adjusted for ICA, or (ICA-WBV)/ICV) and cognitive function in old age (except for LM where there was a negative association) or estimated cognitive change (*g *corrected for NART) (Table 3). Therefore, the association between brain size and cognitive ability in older life is due to the association between current, fluid ability and prior, maximal brain size. Maximal brain volume (ICA) accounted for 6.2% of the variance in *g *in old age, whereas atrophy accounted for < 1%.

**Table 3:** Correlations between cognitive tests and brain volumes corrected for ICA

	**WBV**	**(ICV-WBV)/ICV**	**WBV corr for ICA**	**RFL corr for ICA**	**LFL corr for ICA**	**RTL corr for ICA**	**LTL corr for ICA**	**RAHC corr for ICA**	**LAHC corr for ICA**
**NART**	.23*	.01	-.00	-.01	-.13	-.02	-.00	.08	.14
**RSPM**	.27**	-.11	.11	.05	-.09	.17	.09	.01	.03
**MHT**	.25*	-.05	.05	.11	-.06	.15	.04	.03	.06
**VF**	.15	-.07	.07	.03	-.02	.10	.10	.17	.18
**LM**	-.18	.22*	-.22*	-.01	-.09	-.19	-.20*	-.08	-.04
***g***	.21*	-.03	.03	.07	-.09	.11	.03	.05	.08
***g *corr for NART**	.11	-.04	.04	.09	-.02	.14	.04	.01	.01

* P < .05 ** P < .01

Partial correlation, corrected for sex (df = 96) WBV: whole brain volume, ICA: intracranial area, CCA: corpus callosum area, VV: ventricular volume, RFL: right frontal lobe volume, LFL: left frontal lobe volume, RTL: right temporal lobe volume, LTLV: left temporal lobe volume, RAHC: right amygdalo-hippocampal complex volume, LAHC: left amygdalo-hippocampal complex volume

NART: National Adult Reading Test, RSPM: Raven's Standard Progressive Matrices, MHT: Moray House Test, VF: Verbal Fluency, LM: Logical Memory

*g*: general cognitive factor (The first unrotated principal component of MHT, RSPM, VF and LM, accounting for 51.9% of the total variance: see text.)

*g *corr for NART: estimate of cognitive change (see text)

### Specific cognitive domains and brain regions

There were statistically significant associations between specific brain regions and specific cognitive tests (Table 2), i.e. NART and left AHC; RSPM and temporal lobes; MHT, and right frontal and temporal lobes; VF with AHC. However, when regional volumes were corrected for ICA none of the correlations reached statistical significance (Table 3; similar pattern if corrected for WBV). Adjusting the association between ICA and cognition for specific brain regions attenuates the correlations only to a small degree (e.g. g and ICA correlate at .25, and correcting for RFL, LFL, RTL, LTL, RAHC, LAHC volume attenuate the correlation to .18, .25, .11, .18, .19, .18 respectively). Therefore, the association was due to the relationship between general cognitive ability and overall brain size, not specific cognitive domains and brain regions.

## Discussion

In 107 community-dwelling people aged 75 – 81 years WBV correlated positively with tests of current, 'fluid'-type [10] intelligence. Correlations between regional brain volumes and specific tests became non-significant when adjusted for WBV or ICA. The association between WBV and general cognitive ability was almost wholly attenuated by correcting for ICA. Therefore, associations between brain regions and specific cognitive abilities in (healthy) older age may be largely due to the underlying life-long association between overall brain size and general cognitive ability [14,18].

This replicates the results in a previous study of healthy older men [14]. Our study includes more subjects, both men and women, with a narrow, older, age range [26]. We used minimal exclusion criteria, and therefore our participants should be more typical of the general population than studies selecting healthy participants (e.g. [14]). The prevalence of common diagnoses such as hypertension and diabetes show this group to be comparable to other older volunteer populations [4]. One study of individuals aged over 75 [27] did not find an association between cognition and prior brain size, which may be due to different amounts of white matter damage, or different sample characteristics (e.g. more male participants). Although results from a volunteer population with a limited age range may not be generalisable to the elderly community as a whole, this methodology has important benefits. Since the main correlate with cognitive function is normally age, studying a cohort with a narrow age range allows the relationship between brain MRI data and individual differences in cognitive ability to be investigated [26].

Using volunteers means that there may be selection bias effects we are unaware of, but these are unlikely to produce inflated associations within the cohort. Also, although all subjects scored > 24 on the MMSE, some may be in subclinical stages of dementia, which could affect both brain volumes and cognitive function.

Previous studies have suggested there may be a sex difference in any association between brain size and cognitive ability, with a stronger association for men [15]. In this study including both men and women an association between brain size and cognition persisted when correcting for sex statistically. Our study was not powered to look for sex differences, but emphasises the importance of consideration of sex when investigating brain volumes and cognitive ability.

The concept of 'cerebral reserve' has been introduced to account for differences in cognitive ability in individuals with identical amounts of brain ageing [28]. Cerebral reserve has been characterized by both passive and active components: in passive cerebral reserve, the size of the brain is often used as a proxy for the brain's capacity to withstand the damage accrued by ageing; in active or 'cognitive' cerebral reserve, the brain actively tries to compensate for brain damage using preexisting cognitive processing techniques, or recruiting compensatory approaches. Education or occupation are often used as proxies for cognitive reserve [16]. Cerebral and cognitive reserve are not mutually exclusive concepts, and it is likely that both are involved in protecting the ageing brain from damage accrued with time [28]. The underlying neural mechanisms are suggested to be a) neural reserve, where preexisting neural networks have greater capacity (the passive model) or efficiency, and b) neural compensation, where pathology is compensated for by the recruitment of alternative networks [29]. The finding that cognitive ability in older age correlates with larger brain volume is consistent with the passive model of cerebral reserve [28], and the attenuation of this relationship by education is consistent with the active model [16]. Educational attainment is an important consideration in cohort studies of cognitive ability [30]. It is closely related to childhood cognitive ability (rho = .41 in this study). There is, however, concern in using educational attainment in this cohort born in 1921 – 1926: their educational progress will have been affected by the onset of the Second World War in 1939 (reflected in the median of nine years of formal education). This influence is likely to differ between men and women, and those born in different years, therefore the effect of education in this cohort should be treated with caution, and cannot be generalized to subjects born in other years.

In this cohort maximal brain size (ICA) but not global atrophy was associated with general cognitive ability aged 75–80. The lack of an association between brain atrophy – whether measured by regression or ratio method – [31] and either cognitive ability in older age or cognitive change is interesting. Christensen et al. (2007) also found a correlation between cognitive ability and maximal brain size, but not atrophy, for men but not women [15]. It is possible that atrophy of specific brain regions is associated with decline of specific cognitive abilities [32]. In this study, only logical memory was associated with global atrophy, and in the opposite direction to that expected: i.e. more atrophy correlated with better memory performance. This is unlikely to be a type I error, as similar trends are seen across all the brain regions (uncorrected for ICA), and are stronger in regions that are likely to be associated with memory (e.g. temporal lobe). A negative association between hippocampal volume and memory performance (r ~ 0.25) has been reported in children, adolescents and young adults, whereas the association in older adults is extremely variable [11]. The underlying mechanism for this is unclear, but may due to inadequate neural pruning causing memory to be mediated less effectively in younger people [33]. In older adults, the variability in results may be due to statistical methods of normalizing for age and head size [11,12]. Studies with a narrow age range, such as this one, largely eliminate the influence of age. It is also important that results are presented both raw and adjusted for head (or prior brain) size [11]. Correction for head size is a controversial area [11,12], and we suggest that it should be seen not only as a methodological or statistical issue, but that corrections may in fact obscure the very relationships they are attempting to investigate.

Despite the intuitive appeal of a 'bigger is better'[11] association between brain volume and cognitive performance, structure-cognition associations, where they are found, are not easy to replicate, and are sensitive to the composition of the sample tested, and the cognitive tests used [12]. Differences in white matter volume [18] or grey matter density [34] may be more important than overall volume.

The association between cognitive ability in the eighth decade and maximal brain size – fixed by around age six, and possibly determined by postnatal growth – [35] suggests that research into the biological basis of age-related cognitive change should take a lifecourse perspective [30]. Influences occurring in early life, or even before birth, can affect cognitive ability many decades later [36]. However, it should be noted that the effect size of maximal brain size on cognition is small: ICA accounts for only around 7% of the variance in *g*.

In this healthy cohort, where there is unlikely to be high degrees of atrophy, ICA (as a proxy for WBV) is likely to be close to the actual WBV in earlier life. The association between brain volume and cognitive ability may be different in pathological cognitive impairment. For example, in dementia, correcting for prior brain size may still reveal associations between profound atrophy and impaired cognition [37], and indeed atrophy of specific brain regions such as hippocampus and entorhinal cortex may predict those likely to develop Alzheimer's disease [38]. However, in normal cognitive ageing changes are likely to be more subtle [12]. Cross-sectional studies such as this one, performing multiple comparisons, cannot be conclusive about the strength of associations or the direction of causation. Longitudinal studies over many years are required to accurately establish the relationship between brain structure and function, including cognitive change. Future studies should examine these interrelationships in healthy and cognitively impaired older individuals to establish if there is a point at which the correlation between regional atrophy and specific cognitive tests does become truly significant.

## Conclusion

Individual differences in normal cognitive ageing are more strongly related to prior brain size, and its association with general cognitive ability, than as a consequence of brain atrophy. Researchers and clinicians should therefore be cautious of interpreting global brain atrophy on neuroimaging as contributing to cognitive status in older age without taking into account prior mental ability and brain size.

## Competing interests

The authors declare that they have no competing interests.

## Authors' contributions

SS recruited the participants, performed the cognitive tests, performed the statistical analysis and wrote the first draft of the manuscript. JW established the imaging facility and set up the imaging protocol. CR performed the volumetric analyses. ID, JS and JW conceived of, designed, and supervised the study. CR, ID, JS and JW revised the manuscript critically for important intellectual content, and all authors read and approved the final manuscript.

## Pre-publication history

The pre-publication history for this paper can be accessed here:


